# Cannulation technique and complications in arteriovenous fistulas: a Swedish Renal Registry-based cohort study

**DOI:** 10.1186/s12882-021-02458-z

**Published:** 2021-07-07

**Authors:** Karin Staaf, Anders Fernström, Fredrik Uhlin

**Affiliations:** 1 Department of Nephrology and Department of Health, Medicine and Caring Sciences, Linköping University, SE-581 85 Linköping, Sweden; 2 Department of Health Technologies, Tallinn University of Technology, Tallinn, Estonia

**Keywords:** Area puncture, Blunt needle, Buttonhole, Cannulation, Cannulation-related complications, Hemodialysis, Rope ladder, Sharp needle, Vascular access

## Abstract

**Background:**

The four cannulation techniques, rope ladder (RL), area puncture (AP), buttonhole with blunt needles (BHb), and buttonhole with sharp needles (BHs), affects the arteriovenous fistula (AVF) in different ways. The aim of this study was to describe the relationship between the different cannulation techniques and the occurrence of AVF complications.

**Methods:**

The study was performed as a national registry-based cohort study using data from the Swedish Renal Registry (SRR). Data were collected from January 2014 to October 2019. Seventy of Sweden’s dialysis units participate in the registry. We analyzed a total of 1328 AVFs in this study. The risk of complications was compared between the four different cannulation techniques. The risk of AVF complications was measured by the incidence and incidence rate ratio (IRR). We compared the IRRs of complications between different cannulation techniques.

**Results:**

BHs is the most common cannulation technique in Sweden. It has been used in 55% of the AVFs at some point during their functional patency. BHb (29%), RL (13%), and AP (3%) has been used less. BHb had the lowest risk of complications compared to the other techniques, and a significantly lower risk of stenosis, infiltration, cannulation difficulties, compared to RL and BHs. Cannulation difficulties were significantly more common using AP compared to BHs, and BHb. Infections were not significantly increased using the buttonhole technique.

**Conclusions:**

BHb had the lowest risk of complications. Infections were not significantly increased using the buttonhole technique. Dialysis units with a low infection rate may continue to use the buttonhole technique, as the risk of complications is lower.

**Supplementary Information:**

The online version contains supplementary material available at 10.1186/s12882-021-02458-z.

## Introduction

Individuals with chronic kidney disease (CKD) who require hemodialysis must have functioning access to the blood. There are three ways to create an access: central venous catheter, arteriovenous graft, and arteriovenous fistula (AVF). The AVF is the most common and most preferred access because it has the longest patency and fewest complications [1, 2]. Yet, according to the Dialysis Outcomes and Practice Patterns Study (DOPPS), complications occur in 37% of all new AVFs over the first 6 months. These complications include local and general infections as well as stenosis and thromboses [3]. When the AVF is established, complications decrease. A European follow-up study reported a complication rate of 15.5% at 1 year [4].

AVF complications have different causes and severity, requiring various treatments and preventative approaches [5]. The most common AVF complications were stenosis, thromboses, bleeding, infection, high pressure, aneurysm, flow problems, and steal syndrome [4]. Complications lead to an increased need of care, are time-consuming for the patient, and increased pain, anxiety, and stress [6].

Vascular anatomy, surgical technique, AVF placement, and previous complications, as well as co-morbidity, sex, and age of both the patient and the AVF, affect AVF maturity, occurrence of complications and patency [2, 7]. Other factors that affect the AVF is daily care, time to first cannulation, the cannulation technique used, and the size and angle of the needle inserted [8–11].

Guidelines describe three possible ways to cannulate an AVF: rope ladder (RL), area puncture (AP), and buttonhole (BH). When using RL, the cannulator creates a new puncture site each time. The puncture site is placed 0.5 cm from the last puncture site and the whole length of the AVF is used. Cannulators using AP also create new puncture sites each time, but they place all sites in the same area, rarely larger than 2–3 cm in diameter. When BH is used, the needle is placed in exactly the same cannulation tract, using the same angle each time. To create a tunnel tract, sharp needles are used. When the tract is formed, cannulation can be done with blunt needles [5]. Even though blunt needles are most common using BH, sharp needles might be a long time solution [12–14]. Long-time use with sharp needles will therefore be referred to as buttonhole sharp (BHs) and the cannulation technique using blunt needles as buttonhole blunt (BHb). The different cannulation techniques are used to varying extents worldwide. In the US, RL is the most common technique [15], whereas in Europe, AP is most frequently used (66% vs. RL 30% and BH 6%) [9].

The different cannulation techniques have been investigated and, according to both American and European guidelines, AP should not be used [1, 2, 5] because the risk of complications such as aneurysms and hemorrhage is imminent [16, 17]. Opinions differ when it comes to RL and BH. Studies have shown that the techniques affect patients in different ways. For example, RL leads to more infiltrations and aneurysms but a reduced risk of infection compared to BH [18]. Few randomized trials have been conducted, and most of the conclusions have been drawn from observational studies [2, 19].

A systematic literature review showed that, compared to RL, BH led to fewer interventions related to thromboses and stenosis. BH also led to the formation of fewer aneurysms [20]. In contrast, BH cannulation increases the risk of infections [18]. Studies comparing BHb versus BHs have not reported any significant difference in the incidence of stenosis [12] or any significant difference regarding infections between the two techniques [12, 14]. However, both everyday complications and bleeding between dialysis were more common with BHs than BHb [12].

An optimal cannulation technique remains to be found. Therefore, the outcomes of different cannulation techniques need to be compared in a larger population for a longer period than in previous studies. The population should also include as many dialysis units as possible in order to reduce the effect of local procedures on the outcome. The aim of the present study was to describe the relationship between different AVF cannulation techniques and the occurrence of complications.

## Materials and methods

### Study design

The study was performed as a national registry-based cohort study using data from the Swedish Renal Registry (SRR). SRR includes all adult patients with CKD stage 4–5 in Sweden. Data are continuously registered, including patients’ AVFs and their care. The SRR provides an opportunity to study the entire Swedish hemodialysis population for a long period of time [21]. Registry data were obtained after written approval from the SRR.

### Data collection

Data regarding gender, year of birth, kidney disease, and region were collected for all patients who had a newly created AVF with onset of function during the period 1 January 2014 to 25 October 2019. Data collected regarding AVFs were date of surgery, type, location, functional start (date), cannulation techniques (date range), complications (date), and abandonment (reason and date).

**Fig. 1:**
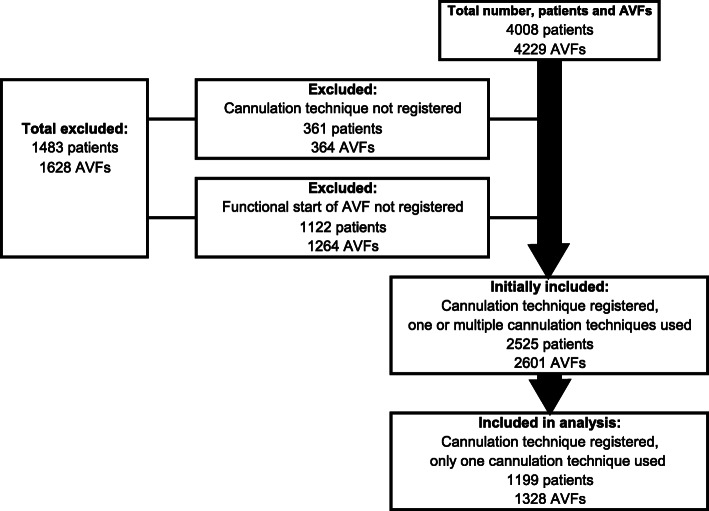
Flow diagram of patients and AVF inclusion in the study

During the period, 4008 individuals with AVFs were registered in the SRR. Patients whose AVFs were abandoned before the first cannulation were excluded, as well as those who lacked a registered cannulation technique (Fig. 1). A total of 2601 AVFs were initially included in the study. Patients in this population had been exposed to one or to several cannulation techniques. After the first analysis the population was reduced to only include patients exposed to a single cannulation technique during patency, i.e. 1328 AVFs. BHs during 1 to 40 days which preceded BHb (to create a tunnel track) was considered as BHb. If BHs was used for longer than 40 days preceding BHb, BHs and BHb were assessed as different techniques. The analysis was based on the included fistulas. The cannulation techniques found in the SRR are BHb, BHs, RL, and AP. The analyses were based on the incidence of AVF complications in relation to the cannulation technique use. Fistula days were calculated from the onset of function to AVF abandonment (occlusion/primary occlusion, AVF ligation, patient refuses use, lost to follow-up, non-function, patient deceased) or end of study.

All registered AVF complications were included. To increase comparability with previous studies and reduce the risk of ambiguities regarding the definition of certain complications, such as aneurysm/pseudoaneurysm and different types of infection, they were grouped into broader categories (Table 1). All defined complications, AVF types and reasons for abandonment are taken from the SRR.

**Table 1:** Definition of complications in the SRR

Complication category	Complication registered in SRR	Definition of complication in SRR
Bleeding	Bleeding	Is registered if it leads to blood transfusion. Reoperation, hospitalization, or prolonged hospitalization
Thrombosis/occlusion	Thrombosis/occlusion	That leads to intervention or AVF abandonment
Stenosis	Stenosis vein Stenosis artery Stenosis vein + vein branches Stenosis artery + vein	Is registered if it leads to angioplasty or if the velocity is 2.5-times increased measured with duplex ultrasound or > 50% reduction in the diameter measured with venography
Infection	Infection Infection - local Infection - general	Local or general infection originating in the AVF and needs treatment with antibiotics
Other	Stenosis central vein Stenosis central vein + other stenosis	The stenosis is called central from the beginning of the vena cephalica in the vena subclavia or proximal of this point; stenosis in central vein registered even though it doesn’t lead to an intervention
	Steal syndrome Vein branches Other	That leads to intervention or AVF abandonment
	Low flow High flow	That leads to intervention or AVF abandonment
Aneurysm	Pseudoaneurysm Aneurysm	That leads to intervention or AVF abandonment
Cannulation difficulty	Cannulation difficulty	That leads to intervention or AVF abandonment
Infiltration	Infiltration	That leads to intervention or AVF abandonment

### Statistical analysis

Incidence and confidence intervals were calculated for each group using the Poisson 95% Confidence Interval using number of events and AVF-days. In between-group comparisons, the incidence rate ratio (IRR) was used [22]. Adjustments for confounders were made using Mantel-Haenszel stratification. Averages were compared using the t-test and ANOVA. Binary outcomes were compared using the χ^2^ test. Due to multiple analyses (six different comparisons) Bonferroni correction was used to correct the statistical significance from *p* <  0.05 to *p* <  0.008. During binary comparison, statistical significance was set to *p* <  0.05. All methods were carried out in accordance with relevant guidelines and regulations.

Microsoft Excel 2010 (Microsoft, USA) was used as a database for collecting and grouping data and calculating the stratification for the Mantel-Haenszel test. Statistical analysis were performed using IBM SPSS Statistics for Windows, version 25 (IBM Corp., Armonk, NY).

### Ethical considerations

The Swedish Ethical Review Authority gave ethical approval (No. 2019–02554) to the study and has waived the need for informed consent.

## Results

The study included 1199 patients and 1328 AVFs (see Fig. 1) that were retrospectively reviewed for 990,405 AVF days. Of the included patients, 29 had two AVFs during the study period. One-third of the group were women, the most common cause of renal failure was diabetes, and the most frequent AVF was radiocephalic fistula. Just over a quarter of the AVFs were located on the patient’s right side (Table 2). The most common technique was BHs. It has been used in 55% of the AVFs. BHb (29%), RL (13%), and AP (3%) were used less. BHb was used for longer periods than both BHs and RL (Table 3).

**Table 2:** AVF characteristics by cannulation technique

	Total		BHs			BHb			RL			AP		
	Number	Percent (%)	Number	Percent (%) of used tech.	Percent (%) of the total	Number	Percent (%) of used tech.	Percent (%) of the total	Number	Percent (%) of used tech.	Percent (%) of the total	Number	Percent (%) of used tech.	Percent (%) of the total
**Causes of ESRD**														
Diabetic nephropathy	327	24.6	181	24.7	55.4	101	26.3	30.9	31	18.6	9.5	14	31.8	4.3
Hypertension	221	16.6	115	15.7	52.0	68	17.7	30.8	30	18.0	13.6	8	18.2	3.6
Other renal disease	266	20.0	149	20.3	56.0	68	17.7	25.6	39	23.4	14.7	10	22.7	3.8
Unknown diagnosis	145	10.9	80	10.9	55.2	36	9.4	24.8	24	14.4	16.6	5	11.4	3.4
Glomerulonephritis	187	14.1	105	14.3	56.1	56	14.6	29.9	22	13.2	11.8	4	9.1	2.1
Polycystic kidney diseases	114	8.6	62	8.5	54.4	36	9.4	31.6	14	8.4	12.3	2	4.5	1.8
Pyelonephritis	58	4.4	35	4.8	60.3	17	4.4	29.3	5	3.0	8.6	1	2.3	1.7
Renal vascular diseases	10	0.8	6	0.8	60.0	2	0.5	20.0	2	1.2	20.0	0	0.0	0.0
**AVF type**														
Brachiocephal	356	26.8	200	27.3	56.2	81	21.1	22.8	67	40.1	18.8	8.0	18.2	2.2
Proximal radiocephal	12	0.9	6	0.8	50.0	5	1.3	41.7	0	0.0	0.0	1.0	2.3	8.3
Radiobasilica	5	0.4	3	0.4	60.0	2	0.5	40.0	0	0.0	0.0	0.0	0.0	0.0
Radiocephal	841	63.3	459	62.6	54.6	278	72.4	33.1	74	44.3	8.8	30.0	68.2	3.6
Ulnar	8	0.6	4	0.5	50.0	2	0.5	25.0	2	1.2	25.0	0.0	0.0	0.0
Other	20	1.5	8	1.1	40.0	4	1.0	20.0	8	4.8	40.0	0.0	0.0	0.0
Brachiobasilica transposed 1 stage	61	4.6	42	5.7	68.9	3	0.8	4.9	12	7.2	19.7	4.0	9.1	6.6
Brachiobasilica transposed 2 stage	20	1.5	9	1.2	45.0	8	2.1	40.0	2	1.2	10.0	1.0	2.3	5.0
Thigh. from *A. femoralis*	3	0.2	0	0.0	0.0	1	0.3	33.3	2	1.2	66.7	0.0	0.0	0.0
Brachiobasilica forearm	2	0.2	2	0.3	100.0	0	0.0	0.0	0	0.0	0.0	0.0	0.0	0.0
**AVF right/left hand side**	369/956	28/72	204/529	28/72		98/286	26/75		48/119	29/71		19/25	43/57	
**Reason for abandonment**														
Thrombosed AVF	64	4.8	34	4.6	53.1	14	3.6	21.9	11	6.6	17.2	5.0	11.4	7.8
Deceased	352	26.5	202	27.6	57.4	85	22.1	24.1	50	29.9	14.2	15.0	34.1	4.3
No function	50	3.8	31	4.2	62.0	7	1.8	14.0	7	4.2	14.0	5.0	11.4	10.0
Primary occlussion	1	0.1	0	0.0	0.0	1	0.3	100.0	0	0.0	0.0	0.0	0.0	0.0
Ligated AVF	56	4.2	36	4.9	64.3	10	2.6	17.9	7	4.2	12.5	3.0	6.8	5.4
Patients choise	7	0.5	7	1.0	100.0	0	0.0	0.0	0	0.0	0.0	0.0	0.0	0.0
Lost to follow up	5	0.4	2	0.3	40.0	1	0.3	20.0	2	1.2	40.0	0.0	0.0	0.0
End of study	793	59.7	421	57.4	53.1	266	69.3	33.5	90	53.9	11.3	16.0	36.4	2.0
**Region in Sweden**														
North	166	8.7	54	7.4	32.5	47	12.2	28.3	15	9	9.0	0	0	0.0
Stockholm	217	16.3	61	8.3	28.1	53	13.8	24.4	101	60.5	46.5	2	4.5	0.9
Southeast	174	13.1	98	13.4	56.3	71	18.5	40.8	1	0.6	0.6	4	9.1	2.3
South	312	23.5	229	31.2	73.4	66	17.2	21.2	8	48	2.6	9	20.5	2.9
Uppsala Örebro	253	19.1	136	18.6	53.8	61	15.9	24.1	33	19.8	13.0	23	52.3	9.1
West	256	19.3	155	21.1	60.5	86	22.4	33.6	9	5.4	3.5	6	13.6	2.3

*Abbreviation*: *BHs* Buttonhole sharp, *BHb* Buttonhole blunt, *RL* Rope ladder, *AP* Area puncture

**Table 3:** AVF characteristics by cannulation technique

	Total		BHs		BHb		RL		AP	
	Number	Percent	Number	Percent	Number	Percent	Number	Percent	Number	Percent
**Female/male**	395/933	30/70	238/495	32/68	86/298	22/78	49/118	29/71	22/22	50/50
**Age (mean. ± SD)**	64.3 (±14.9)		65.0 (±14.8)		61.3 (±15.6)		67.6 (±15.1)		65.7 (±14.9)	
**Number of AVF days/AVF (mean. ± SD)**	746 (± 532)		723 (±544)		867 (±534)		623 (±413)		524 (±504)	
**Number of AVF days (number. %)**	990,405	100	530,322	54	332,944	34	104,041	11	23,098	2
**Number of AVFs using the technique (number. %)**	1328	100	733	55	384	29	167	13	44	3

*Abbreviations*: *SD* Standard Deviation, *BHs* Buttonhole sharp, *BHb* Buttonhole blunt, *RL* Ropeladder, *AP* Area puncture

**Fig. 2:**
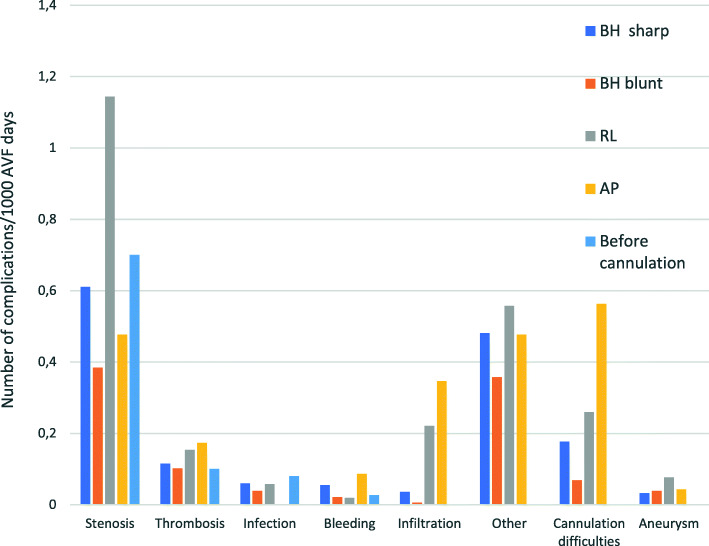
Number of complications per 1000 AVF-days for different cannulation techniques according to AVF complication. Before cannulation is the time from the creation of the AVF until the cannulation began

We found a difference in the occurrence of complications when comparing cannulation techniques, and differences between the various types of complications. Stenosis is the most common complication for all cannulation techniques and before exposure to needles. When cannulation techniques are compared against each other, BHb has the lowest risk of complications compared to the other techniques (Fig. 2 and Table 4).

**Table 4:** Comparisons and statistical analysis of the number of complications with the different cannulation techniques

*n* = 1328	No of complic. /1000 AVF days	95% CI	No of complic /1000 AVF days	95% CI	IRR	95% CI	***P*** value
	**BHs**		**BHb**				
**Stenosis**	**0.61**	**0.55 to 0.68**	**0.38**	**0.32 to 0.46**	**1.59**	**1.29 to 1.97**	**< 0.001**
**Thrombosis**	0.12	0.09 to 0.15	0.10	0.07 to 0.14	1.13	0.73 to 1.77	0.58
**Infection**	0.06	0.04 to 0.09	0.04	0.02 to 0.07	1.55	0.79 to 3.21	0.18
**Bleeding**	0.05	0.04 to 0.08	0.02	0.01 to 0.04	2.60	1.12 to 7.03	0.02
**Infiltration**	0.04	0.02 to 0.06	0.01	0.001 to 0.02	5.96	1.44 to 52.81	0.01
**Other**	0.48	0.42 to 0.54	0.36	0.3 to 0.43	1.35	1.08 to 1.69	0.01
**Cannulation difficulty**	**0.18**	**0.14 to 0.22**	**0.07**	**0.04 to 0.1**	**2.57**	**1.61 to 4.24**	**< 0.001**
**Aneurysm**	0.03	0.02 to 0.05	0.04	0.02 to 0.07	0.82	0.38 to 1.84	0.59
	**BHs**		**RL**				
**Stenosis**	**0.61**	**0.55 to 0.68**	**1.14**	**0.95 to 1.37**	**0.53**	**0.43 to 0.66**	**< 0.001**
**Thrombosis**	0.12	0.09 to 0.15	0.15	0.09 to 0.25	0.75	0.43 to 1.39	0.30
**Infection**	0.06	0.04 to 0.09	0.06	0.02 to 0.13	1.05	0.43 to 3.06	0.92
**Bleeding**	0.05	0.04 to 0.08	0.02	0.002 to 0.07	2.84	0.72 to 24.60	0.13
**Infiltration**	**0.04**	**0.02 to 0.06**	**0.22**	**0.14 to 0.33**	**0.16**	**0.08 to 0.31**	**< 0.001**
**Other**	0.48	0.42 to 0.54	0.56	0.42 to 0.72	0.86	0.65 to 1.17	0.31
**Cannulation difficulty**	0.18	0.14 to 0.22	0.26	0.17 to 0.38	0.68	0.44 to 1.09	0.08
**Aneurysm**	0.03	0.02 to 0.05	0.08	0.03 to 0.15	0.42	0.17 to 1.12	0.04
	**BHs**		**AP**				
**Stenosis**	0.61	0.55 to 0.68	0.48	0.24 to 0.85	1.29	0.71 to 2.60	0.41
**Thrombosis**	0.12	0.09 to 0.15	0.17	0.05 to 0.44	0.66	0.25 to 2.52	0.42
**Infection**	0.06	0.04 to 0.09	0	0 to 0.2	–	–	0.24
**Bleeding**	0.05	0.04 to 0.08	0.09	0.01 to 0.31	0.63	0.16 to 5.46	0.53
**Infiltration**	**0.04**	**0.02 to 0.06**	**0.35**	**0.15 to 0.68**	**0.10**	**0.04to 0.27**	**< 0.001**
**Other**	0.48	0.42 to 0.54	0.48	0.24 to 0.85	1.01	0.55 to 2.05	0.98
**Cannulation difficulty**	**0.18**	**0.14 to 0.22**	**0.56**	**0.3 to 0.96**	**0.31**	**0.18 to 0.61**	**< 0.001**
**Aneurysm**	0.03	0.02 to 0.05	0.04	0.001 to 0.24	0.74	0.12 to 30.94	0.77
	**BHb**		**RL**				
**Stenosis**	**0.38**	**0.32 to 0.46**	**1.14**	**0.95 to 1.37**	**0.34**	**0.26 to 0.44**	**< 0.001**
**Thrombosis**	0.10	0.07 to 0.14	0.15	0.09 to 0.25	0.66	0.36 to 1.29	0.17
**Infection**	0.04	0.02 to 0.07	0.06	0.02 to 0.13	0.68	0.24 to 2.17	0.43
**Bleeding**	0.02	0.01 to 0.04	0.02	0.002 to 0.07	1.09	0.21 to 10.79	0.91
**Infiltration**	**0.01**	**0.001 to 0.02**	**0.22**	**0.14 to 0.33**	**0.03**	**0.003 to 0.11**	**< 0.001**
**Other**	0.36	0.3 to 0.43	0.56	0.42 to 0.72	0.64	0.46 to 0.89	0.01
**Cannulation difficulty**	**0.07**	**0.04 to 0.1**	**0.26**	**0.17 to 0.38**	**0.27**	**0.15 to 0.48**	**< 0.001**
**Aneurysm**	0.04	0.02 to 0.07	0.08	0.03 to 0.15	0.51	0.20 to 1.41	0.12
	**AP**		**BHb**				
**Stenosis**	0.48	0.24 to 0.85	0.38	0.32 to 0.46	1.24	0.60 to 2.29	0.49
**Thrombosis**	0.17	0.05 to 0.44	0.10	0.07 to 0.14	1.70	0.44 to 4.75	0.31
**Infection**	0	0 to 0.2	0.04	0.02 to 0.07	0.00	0.00 to 4.73	0.34
**Bleeding**	0.09	0.01 to 0.31	0.02	0.01 to 0.04	4.12	0.42 to 21.63	0.06
**Infiltration**	**0.35**	**0.15 to 0.68**	**0.01**	**0.001 to 0.02**	**57.66**	**11.51 to 557.34**	**< 0.001**
**Other**	0.48	0.24 to 0.85	0.36	0.3 to 0.43	1.33	0.65 to 2.47	0.36
**Cannulation difficulty**	**0.56**	**0.3 to 0.96**	**0.07**	**0.04 to 0.1**	**8.15**	**3.79 to 16.77**	**< 0.001**
**Aneurysm**	0.04	0.001 to 0.24	0.04	0.02 to 0.07	1.11	0.03 to 7.38	0.92
	**AP**		**RL**				
**Stenosis**	**0.48**	**0.24 to 0.85**	**1.14**	**0.95 to 1.37**	**0.42**	**0.20 to 0.77**	**0.004**
**Thrombosis**	0.17	0.05 to 0.44	0.15	0.09 to 0.25	1.13	0.27 to 3.49	0.83
**Infection**	0	0 to 0.2	0.06	0.02 to 0.13	0.00	0.00 to 3.83	0.25
**Bleeding**	0.09	0.01 to 0.31	0.02	0.002 to 0.07	4.50	0.33 to 62.14	0.10
**Infiltration**	0.35	0.15 to 0.68	0.22	0.14 to 0.33	1.57	0.61 to 3.63	0.27
**Other**	0.48	0.24 to 0.85	0.56	0.42 to 0.72	0.85	0.40 to 1.64	0.63
**Cannulation difficulty**	0.56	0.3 to 0.96	0.26	0.17 to 0.38	2.17	1.03 to 4.35	0.02
**Aneurysm**	0.04	0.001 to 0.24	0.08	0.03 to 0.15	0.56	0.01 to 4.20	0.58

As the significance level is adjusted only *p*-values < 0.008 are bold. *Abbreviation*: *CI* Confidence interval, *IRR* Incidence risk ratio, *CI* Confidence interval, *IRR* Incidence risk ratio, *BHs* Buttonhole sharp, *BHb* Buttonhole blunt, RL Rope ladder, *AP* Area puncture

BHb has a significantly lower risk of stenosis, infiltration and cannulation difficulties, compared to RL and a significantly lower risk of stenosis and cannulation difficulties compared to BHs. Cannulation difficulties are significantly more common with AP than BHb and BHs. With regard to infections, there was not a significant difference between BHs and BHb compared to RL (Table 4).

No differences were found in the majority of the values when controlling for the confounding factors gender, diabetes, and right-sided AVF (Table S2).

The risk of having a complication was increased, but not significantly, among women and those with right-sided AVF. Patients with diabetes and those older than 70 years had an increased risk of stenosis when using BHs and BHb. On the other hand, the infection risk was significantly decreased in patients older than 70 years when using BHb (Table S1).

We found a significant difference (*P* <  0.001) between the amount of patients who did not have complications (57.1%) compared to those who had complications (42.9%), regardless of cannulation technique. Those who had least one complication were significantly older (65.2 compared to 63.5 years of age, *P* = 0.04). Those who had at least one complication, in the initial group of 2601 AVFs (see Fig. 1), were exposed to significantly more cannulation techniques (1.8 ± 1.025) compared to those who had no complications (1.6 ± 0.83; *P* <  0.001).

## Discussion

This study indicates that there are disparities in complication frequency between different cannulation techniques. BHb has the lowest risk of complications compared to BHs, RL, and AP. Registry data from the SRR made it possible to include a large population from 67 different dialysis units in Sweden. This provided us an opportunity to study the relationship between cannulation techniques and complications, but also an opportunity to examine the long-term consequences of the various cannulation techniques. BHs and BHb were the most commonly used techniques, followed by RL. AP was not used extensively (3%) in this population. There are also local variations in both choice of cannulation technique, age and routines that may have affected the result. These variations are both known (Table 3) and unknown (for example needle or bevel direction) as they were not registered in the SRR.

Only 9% of the 4229 AVFs were excluded because of lack of registered cannulation technique (see Fig. 1). This indicates that registration in the SRR is quite good, which is also confirmed by the registry validations [23, 24]. Thirty percent of AVFs were excluded due to short AVF patency and abandonment before cannulation was started. This can be compared to the numbers of abandoned AVFs from previous studies, with the numbers matching quite well [2].

AVF-related infections are one of the complications that lacked significant differences in prevalence, regardless of cannulation technique. Two systematic literature reviews have examined the frequency of infection related to AVF and cannulation technique. They reported a trend towards an increased infection rate when using the BH technique [18, 20]. However, the studies included in these reviews were relatively small and differed in the duration of follow-up and hygiene routines, such as the type of disinfectant used, when disinfection was performed in relation to removal of the scab, and how the scab was removed [25–27].

The number of AVF-related infections in this study were low compared to previous studies [15, 26, 28]. When comparing our study to the frequency reported by a study from Belgium [29], the infection rate was in rough agreement regardless of cannulation technique.

What could be suspected is that not all AVF infections are registered. However, the low infection rate in our study is in line with the frequency of AVF infections found in a review of medical records from southern Sweden [12]. The low number of total infections in the Swedish dialysis population also corresponds well with the low total mortality rate due to infections. In Sweden, the total infection-related mortality among patients in need of dialysis is 3% [30]. A study from the Netherlands, Canada, Norway, Spain, France, and Turkey reported a total infection mortality rate of 6% in the dialysis population [31], and a Danish study reported a total infection mortality rate of 4.1% [32]. The occurrence of interventions due to stenosis [33] and hemorrhage [28] also seems to be in the same magnitude as in previous studies, therefore we assume that the incidence of infections also has been reported to the same extent.

Studies have previously reported that strict adherence to the hygiene routines is important when BH is used [28, 34]. The low rate of AVF infections in this study may depend on well-established hygiene protocols and good adherence to these routines in the studied population. It may also be easier to remember performing all of the steps in the hygiene protocol if the majority of patients are cannulated using the BH technique compared to if only a few in the unit use this technique. Previous studies indicate that compliance with hygiene routines can easily fail [34, 35].

This study indicates that diabetes slightly increased (not significant) the risk of AVF-related infections. Right-sided AVF also emerged as a factor that perhaps increased the risk of AVF infection in BHs. The reason is unclear. It may be more difficult to wash the dominant arm properly between dialysis sessions. Previous studies have demonstrated that skin bacteria, such as *Staphylococcus aureus*, cause the majority of AVF infections [15, 36]. If this is correct and poor hygiene on the dominant arm increases the risk of infections, then good personal hygiene is important for avoiding AVF-related infections. Kaplowitz et al. previously stated that the degree of personal hygiene affects the outcome of this type of infection [37]. As other complications also are more common in the AVFs in right arms (see Table S1) there might be a correlation between these complications and their interventions and the risk of AVF infections.

Even though this study did not find a significant difference in infection frequency between RL and BH, other studies have reported such a difference [15, 28]. This suggests that certain hygiene routines affect the frequency. However, as long as the cause of the increased infection rates is unknown, more research is warranted. Units using BH that have a low infection rate should continue to adhere to their hygiene routines but also register and evaluate their infection rates.

Regardless of cannulation technique, stenosis was the most common type of complication. This finding confirms the tendency in previous studies of BHb patients having fewer interventions for stenosis and thrombosis [18]. Stenosis is more common in patients who receive an AVF using BH technique and have diabetes (see Table S1). Stenosis is also more common among patients aged over 70 years using BHb, BHs and RL. Therefore, it is likely that the development of stenosis is generally more common in the elderly. It is important to have in mind that stenosis do have other risk factors than the choice of cannulation technique.

Hemorrhages were not, more likely when AP was used. Perhaps this was a result of the small size of patients using this technique.

Previous studies have shown that the development of aneurysms and pseudoaneurysms is more likely using both AP and RL compared to BHb [34, 38]. This was not confirmed in this study. Notably, however, the only aneurysms/pseudoaneurysms that are registered are those requiring some kind of treatment. Untreated dilated AVFs will remain unrecorded, and the true incidence of aneurysm related to the cannulation technique will be left to future studies to explore.

Surprisingly, AP caused more cannulation difficulties than other techniques. AP is the most frequently used technique in Europe, as it causes few acute complications, such as infiltrations and cannulation difficulties, and is easy to use [10]. The cannulation difficulties in this study are not those that nurses and patients encounter in everyday life. Cannulation difficulties registered in the SRR lead to some kind of treatment. Therefore, it is likely that AP is chosen when the AVFs are small and thin in order to widen the AVF [38]. When the effect of this intervention fails, the AVF has to be treated with, for example, an angioplasty to prolong its patency.

Additional factors that affect the incidence of complications are older age, female gender, and having diabetes, which results in an increased risk of developing complications [12, 39]. In addition, in the initial group of 2601 AVF (see Fig. 1) s, patients who had one or more complications changed the cannulation technique several times. Therefore, the occurrence of a complication is likely to affect the choice of cannulation technique.

The different cannulation techniques affect the incidence of complications to varying degrees. Therefore, the choice of cannulation technique can result in an increased or decreased risk of complications. Guidelines primarily advocate RL because of the low risk of infections. According to the same guidelines, BH should only be used in those with a short cannulation segment [1, 2, 40]. The present study shows that it is possible to use BH, as well as RL, for a long period with an equivalent infection frequency. Therefore, it is reasonable to consider the increased risk of all types of complications when choosing a cannulation technique. Viecelli et al. found that both patients and health professionals think that a functioning AVF is the most important issue [41]. If it is possible to prevent stenosis, infiltrations and cannulation difficulties by choosing a particular cannulation technique, it is possible to reduce suffering from painful and time-consuming complications and simultaneously reduce expensive interventions. This is also in line with the person-centred approach gaining ground in health care.

The present study has several limitations. First, as registration is done retrospectively and manually by participating dialysis units, there is uncertainty whether data are transmitted correctly. Another limitation is that several of the observed complications are difficult to define. Therefore, it is uncertain whether all complications that occurred were properly registered. It is also unclear what type of complications are hidden behind the category “other” in the SRR. Complications may be placed there instead of under the proper category. Several of the complications also have other confounding factors than those included in SRR, for example not registered everyday complications, direction of the needle or indications and routines for choosing one cannulation technique over the other.

Future studies in this field should examine how a cannulation technique is chosen for the individual patient. For example, is the choice of cannulation technique influenced by previous complications? It would also be valuable to investigate each cannulation technique in a prospective study to determine their impact on everyday complications, such as oozing, prolonged post-dialysis bleeding, and AVF maturation. Additional studies are also of interest regarding the optimal hygiene routine during cannulation. For example, does it matter what type of disinfectant is used and how it is applied?

## Conclusions

The cannulation technique with the fewest complications in this study is BHb. BH cannulation does not necessarily increase the risk of AVF infection. Both BHs and BHb can be used with an incidence of infections in the same magnitude as RL and AP. Units that already have a low infection rate may continue using BH. In this way, it is possible to reduce cannulation-related complications, increase quality of life for the patients, and decrease socio-economic burden on the healthcare organization.

## Supplementary Information

**Additional file 1: Table S1.** AVFs distributed by cannulation technique and subgroups by gender, side, diabetes status, and age. **Table S2.** Comparisons and statistical analysis of the number of complications with the different cannulation techniques.

## Data Availability

*The data underlying this article cannot be shared publicly due to* the privacy of individuals that participated in the study. The data are available from Swedish Renal Registry (SRR). Restrictions apply to the availability of these data, which were used under license for this study. Data are available at https://www.medscinet.net/snr/forskning.aspx with the permission of SRR.

## Abbreviations

AP: Area puncture
AVF: Arteriovenous fistula
BH: Buttonhole
BHb: Buttonhole blunt
BHs: Buttonhole sharp
CI: Confidence Interval
CKD: Chronic Kidney Disease
ESRD: End Stage Renal Disease
IRR: Incidence Rate Ratio
MH: Mantel-Haenszel
RL: Ropeladder
SD: Standard deviation
SRR: Swedish Renal Registry
