# Supplementary material for: Cannulation technique and complications in arteriovenous fistulas: a Swedish Renal Registry-based cohort study
Source: BMC Nephrol. 2021 Jul 7;22:256. doi: 10.1186/s12882-021-02458-z (PMC8265150; doi:10.1186/s12882-021-02458-z)
Supplement: Supplementary file 1 — Additional file 1: Table S1. AVFs distributed by cannulation technique and subgroups by gender, side, diabetes status, and age. Table S2. Comparisons and statistical analysis of the number of complications with the different cannulation techniques. [file 12882_2021_2458_MOESM1_ESM.docx]

| Table S 1. AVFs distributed by cannulation technique and subgroups by gender, side, diabetes status, and age. | | | | | | | | | |
| --- | --- | --- | --- | --- | --- | --- | --- | --- | --- |
|  | **Buttonhole sharp needle** | | | | | | | |  |
|  | **Compli-cations** | **AVF-days** | **Complications /1000 AVF days** | **Compli-cations** | **AVF-days** | **Complications /1000 AVF days** | ***P* value** | **IRR (95% CI)** | |
|  | **Women** | | | **Men** | | |  |  |  |
| **Stenosis** | 84 | 168301 | 0,50 | 240 | 362021 | 0,66 | 0,02 | 0,75 | (0.58 to 0.97) |
| **Thrombosis** | 27 | 168301 | 0,16 | 34 | 362021 | 0,09 | 0,04 | 1,71 | (0.99 to 2.92) |
| **Infection** | 10 | 168301 | 0,06 | 22 | 362021 | 0,06 | 1,00 | 0,98 | (0.41 to 2.15) |
| **Bleeding** | 12 | 168301 | 0,07 | 17 | 362021 | 0,05 | 0,28 | 1,52 | (0.66 to 3.37) |
| **Infiltration** | 9 | 168301 | 0,05 | 10 | 362021 | 0,03 | 0,16 | 1,94 | (0.70 to 5.30) |
| **Other** | 85 | 168301 | 0,51 | 170 | 362021 | 0,47 | 0,58 | 1,08 | (0.82 to 1.40) |
| **Cannulation problem** | 43 | 168301 | 0,26 | 51 | 362021 | 0,14 | **0,005** | 1,81 | (1.18to 2.78) |
| **Aneurysm** | 6 | 168301 | 0,04 | 11 | 362021 | 0,03 | 0,74 | 1,17 | (0.36 to 3.46) |
|  | **AVF right** | | | **AVF left** | | |  |  |  |
| **Stenosis** | 105 | 144489 | 0,73 | 219 | 385833 | 0,57 | 0,04 | 1,28 | (1.01 to 1.62) |
| **Thrombosis** | 21 | 144489 | 0,15 | 40 | 385833 | 0,10 | 0,22 | 1,40 | (0,79 to 2.44) |
| **Infection** | 14 | 144489 | 0,10 | 18 | 385833 | 0,05 | 0,05 | 2,08 | (0.96 to 4.42) |
| **Bleeding** | 8 | 144489 | 0,06 | 21 | 385833 | 0,05 | 0,94 | 1,02 | (0.39 to 2.39) |
| **Infiltration** | 9 | 144489 | 0,06 | 10 | 385833 | 0,03 | 0,07 | 2,40 | (0.86 to 6.58) |
| **Other** | 81 | 144489 | 0,56 | 174 | 385833 | 0,45 | 0,11 | 1,24 | (0.94 to 1.63) |
| **Cannulation problem** | 30 | 144489 | 0,21 | 64 | 385833 | 0,17 | 0,31 | 1,25 | (0.78 to 1.96) |
| **Aneurysm** | 7 | 144489 | 0,05 | 10 | 385833 | 0,03 | 0,22 | 1,87 | (0.60 to 5.44) |
|  | **Diabetes** | | | **No diabetes** | | |  |  |  |
| **Stenosis** | 104 | 110198 | 0,94 | 220 | 420124 | 0,52 | **< 0,0001** | 1,80 | (1.41 to 2.29) |
| **Thrombosis** | 19 | 110198 | 0,17 | 42 | 420124 | 0,10 | 0,06 | 1,72 | (0.95 to 3.03) |
| **Infection** | 5 | 110198 | 0,05 | 27 | 420124 | 0,06 | 0,50 | 0,71 | (0.21 to 1.86) |
| **Bleeding** | 3 | 110198 | 0,03 | 26 | 420124 | 0,06 | 0,16 | 0,44 | (0.09 to 1.44) |
| **Infiltration** | 3 | 110198 | 0,03 | 16 | 420124 | 0,04 | 0,63 | 0,71 | (0.13 to 2.50) |
| **Other** | 58 | 110198 | 0,53 | 197 | 420124 | 0,47 | 0,44 | 1,12 | (0.82 to 1.51) |
| **Cannulation problem** | 26 | 110198 | 0,24 | 68 | 420124 | 0,16 | 0,11 | 1,46 | (0.89 to 2.32) |
| **Aneurysm** | 3 | 110198 | 0,03 | 14 | 420124 | 0,03 | 1,00 | 0,82 | (0.15 to 2.93) |
|  | **Age ≥ 70 years old** | | | **Age < 70 years old** | | |  |  |  |
| **Stenosis** | 163 | 219377 | 0,74 | 161 | 310945 | 0,52 | **0,001** | 1,44 | (1.15 to 1.80) |
| **Thrombosis** | 16 | 219377 | 0,07 | 45 | 310945 | 0,14 | 0,01 | 0,50 | (0.27 to 0.91) |
| **Infection** | 8 | 219377 | 0,04 | 24 | 310945 | 0,08 | 0,06 | 0,47 | (0.18 to 1.09) |
| **Bleeding** | 18 | 219377 | 0,08 | 11 | 310945 | 0,04 | 0,03 | 2,32 | (1.04 to 5.43) |
| **Infiltration** | 13 | 219377 | 0,06 | 6 | 310945 | 0,02 | 0,02 | 3,07 | (1.09 to 9.85) |
| **Other** | 109 | 219377 | 0,50 | 146 | 310945 | 0,47 | 0,65 | 1,06 | (0.82 to 1.37) |
| **Cannulation problem** | 42 | 219377 | 0,19 | 52 | 310945 | 0,17 | 0,51 | 1,14 | (0.74 to 1.75) |
| **Aneurysm** | 3 | 219377 | 0,01 | 14 | 310945 | 0,05 | 0,05 | 0,30 | (0.06 to 1.09) |
|  | **Buttonhole blunt needle** | | | | | | | |  |
|  | **Compli-cations** | **AVF-days** | **Complications /1000 AVF days** | **Compli-cations** | **AVF-days** | **Complications /1000 AVF days** | ***P* value** | **IRR (95% CI)** | |
|  | **Women** | | | **Men** | | |  |  |  |
| **Stenosis** | 29 | 77701 | 0,37 | 99 | 255243 | 0,39 | 0,87 | 0,96 | (0.61 to 1.47) |
| **Thrombosis** | 6 | 77701 | 0,08 | 28 | 255243 | 0,11 | 0,45 | 0,70 | (0.24 to 1.73) |
| **Infection** | 1 | 77701 | 0,01 | 12 | 255243 | 0,05 | 0,19 | 0,27 | (0.01 to 1.85) |
| **Bleeding** | 5 | 77701 | 0,06 | 2 | 255243 | 0,01 | 0,01 | 8,21 | (1,34 to 86,24) |
| **Infiltration** | 1 | 77701 | 0,01 | 1 | 255243 | 0,004 | 0,47 | 3,28 | (0.04 to 257.86) |
| **Other** | 44 | 77701 | 0,57 | 75 | 255243 | 0,29 | **0,001** | 1,93 | (1.30 to 2.83) |
| **Cannulation problem** | 6 | 77701 | 0,08 | 17 | 255243 | 0,07 | 0,73 | 1,16 | (0.37 to 3.08) |
| **Aneurysm** | 6 | 77701 | 0,08 | 7 | 255243 | 0,03 | 0,08 | 2,82 | (0.78 to 9.78) |
|  | **AVF right** | | | **AVF left** | | |  |  |  |
| **Stenosis** | 44 | 75243 | 0,58 | 84 | 257701 | 0,33 | **0,002** | 1,794 | 1.22 to 2.61) |
| **Thrombosis** | 6 | 75243 | 0,08 | 28 | 257701 | 0,11 | 0,51 | 0,734 | (0.25 to 1.81) |
| **Infection** | 3 | 75243 | 0,04 | 10 | 257701 | 0,04 | 0,93 | 1,027 | (0,18 to 3,99) |
| **Bleeding** | 3 | 75243 | 0,04 | 4 | 257701 | 0,02 | 0,25 | 2,569 | (0.38 to 15.18) |
| **Infiltration** | 0 | 75243 | 0,00 | 2 | 257701 | 0,01 | 0,44 | - | (0.00 to 18.24) |
| **Other** | 36 | 75243 | 0,48 | 83 | 257701 | 0,32 | 0,05 | 1,486 | (0.98 to 2.22) |
| **Cannulation problem** | 8 | 75243 | 0,11 | 15 | 257701 | 0,06 | 0,18 | 1,827 | (0.67 to 4.59) |
| **Aneurysm** | 4 | 75243 | 0,05 | 9 | 257701 | 0,03 | 0,49 | 1,522 | (0.34 to 5.45) |
|  | **Diabetes** | | | **No diabetes** | | |  |  |  |
| **Stenosis** | 48 | 75374 | 0,64 | 80 | 257570 | 0,31 | **0,0002** | 2,05 | (1.40 to 2.97) |
| **Thrombosis** | 5 | 75374 | 0,07 | 29 | 257570 | 0,11 | 0,27 | 0,59 | (0.18 to 1.54) |
| **Infection** | 6 | 75374 | 0,08 | 7 | 257570 | 0,03 | 0,07 | 2,93 | (0.81 to 10.18) |
| **Bleeding** | 2 | 75374 | 0,03 | 5 | 257570 | 0,02 | 0,69 | 1,37 | (0.13 to 8.35) |
| **Infiltration** | 1 | 75374 | 0,01 | 1 | 257570 | 0,00 | 0,45 | 3,42 | (0.04 to 268.24) |
| **Other** | 43 | 75374 | 0,57 | 76 | 257570 | 0,30 | **0,001** | 1,93 | (1.30 to 2.85) |
| **Cannulation problem** | 6 | 75374 | 0,08 | 17 | 257570 | 0,07 | 0,68 | 1,21 | (0.39 to 3.21) |
| **Aneurysm** | 3 | 75374 | 0,04 | 10 | 257570 | 0,04 | 0,93 | 1,03 | (0.18 to 3.98) |
|  | **Age ≥ 70 years old** | | | **Age < 70 years old** | | |  |  |  |
| **Stenosis** | 69 | 219377 | 0,31 | 59 | 310945 | 0,19 | **0,004** | 1,66 | (1.15 to 2.39) |
| **Thrombosis** | 9 | 219377 | 0,04 | 25 | 310945 | 0,08 | 0,08 | 0,51 | (0.21 to 1.13) |
| **Infection** | 0 | 219377 | 0,00 | 13 | 310945 | 0,04 | **0,003** | - | (0.00 to 0.46) |
| **Bleeding** | 6 | 219377 | 0,03 | 1 | 310945 | 0,00 | 0,02 | 8,50 | (1.03 to 391.18) |
| **Infiltration** | 0 | 219377 | 0,00 | 2 | 310945 | 0,01 | 0,23 | - | (0.00 to 7.55) |
| **Other** | 63 | 219377 | 0,29 | 56 | 310945 | 0,18 | 0,01 | 1,59 | (1.09 to 2.33) |
| **Cannulation problem** | 13 | 219377 | 0,06 | 10 | 310945 | 0,03 | 0,15 | 1,84 | (0.75 to 4.69) |
| **Aneurysm** | 3 | 219377 | 0,01 | 10 | 310945 | 0,03 | 0,19 | 0,43 | (0.08 to 1.65) |
|  | **Ropeladder** | | | | | | | |  |
|  | **Compli-cations** | **AVF-days** | **Complications /1000 AVF days** | **Compli-cations** | **AVF-days** | **Complications /1000 AVF days** | ***P* value** | **IRR (95% CI)** | |
|  | **Women** | | | **Men** | | |  |  |  |
| **Stenosis** | 27 | 30232 | 0,89 | 92 | 73809 | 1,25 | 0,12 | 0,72 | (0.45 to 1.11) |
| **Thrombosis** | 10 | 30232 | 0,33 | 6 | 73809 | 0,08 | 0,01 | 4,07 | (1.34 to 13.62) |
| **Infection** | 1 | 30232 | 0,03 | 5 | 73809 | 0,07 | 0,57 | 0,49 | (0.01 to 4.37) |
| **Bleeding** | 2 | 30232 | 0,07 | 0 | 73809 | 0,00 | 0,03 | - | - |
| **Infiltration** | 10 | 30232 | 0,33 | 13 | 73809 | 0,18 | 0,14 | 1,88 | (0.74 to 4.64) |
| **Other** | 12 | 30232 | 0,40 | 46 | 73809 | 0,62 | 0,16 | 0,64 | (0.31 to 1.22) |
| **Cannulation problem** | 13 | 30232 | 0,43 | 14 | 73809 | 0,19 | 0,04 | 2,27 | (0.98 to 5.20) |
| **Aneurysm** | 1 | 30232 | 0,03 | 7 | 73809 | 0,09 | 0,34 | 0,35 | (0.01 to 2.71) |
|  | **AVF right** | | | **AVF left** | | |  |  |  |
| **Stenosis** | 36 | 28936 | 1,24 | 83 | 75105 | 1,11 | 0,55 | 1,13 | (0.74 to 1.68) |
| **Thrombosis** | 6 | 28936 | 0,21 | 10 | 75105 | 0,13 | 0,40 | 1,56 | (0.47 to 4.73) |
| **Infection** | 4 | 28936 | 0,14 | 2 | 75105 | 0,03 | 0,06 | 5,19 | (0.74 to 57.39) |
| **Bleeding** | 1 | 28936 | 0,03 | 1 | 75105 | 0,01 | 0,56 | 2,60 | (0.03 to 203.74) |
| **Infiltration** | 6 | 28936 | 0,21 | 17 | 75105 | 0,23 | 1,00 | 0,92 | (0.30 to 2.44) |
| **Other** | 11 | 28936 | 0,38 | 47 | 75105 | 0,63 | 0,13 | 0,61 | (0.28 to 1.19) |
| **Cannulation problem** | 10 | 28936 | 0,35 | 17 | 75105 | 0,23 | 0,30 | 1,53 | (0.62 to 3.53) |
| **Aneurysm** | 1 | 28936 | 0,03 | 7 | 75105 | 0,09 | 0,37 | 0,37 | (0.01 to 2.89) |
|  | **Diabetes** | | | **No diabetes** | | |  |  |  |
| **Stenosis** | 13 | 14748 | 0,88 | 106 | 89293 | 1,19 | 0,31 | 0,74 | (0.38 to 1.33) |
| **Thrombosis** | 1 | 14748 | 0,07 | 15 | 89293 | 0,17 | 0,40 | 0,40 | (0.01 to 2.62) |
| **Infection** | 1 | 14748 | 0,07 | 5 | 89293 | 0,06 | 0,80 | 1,21 | (0.03 to 10.82) |
| **Bleeding** | 0 | 14748 | 0,00 | 2 | 89293 | 0,02 | 0,57 | - | (0.00 to 32.24) |
| **Infiltration** | 6 | 14748 | 0,41 | 17 | 89293 | 0,19 | 0,13 | 2,14 | (0.69 to 5.68) |
| **Other** | 13 | 14748 | 0,88 | 45 | 89293 | 0,50 | 0,09 | 1,75 | (0.87 to 3.30) |
| **Cannulation problem** | 11 | 14748 | 0,75 | 16 | 89293 | 0,18 | **0,001** | 4,16 | (1.75 to 9.55) |
| **Aneurysm** | 1 | 14748 | 0,07 | 7 | 89293 | 0,08 | 1,00 | 0,86 | (0.019 to 6.73) |
|  | **Age ≥ 70 years old** | | | **Age < 70 years old** | | |  |  |  |
| **Stenosis** | 83 | 59350 | 1,40 | 36 | 44691 | 0,81 | **0,005** | 1,74 | (1.16 to 2.64) |
| **Thrombosis** | 9 | 59350 | 0,15 | 7 | 44691 | 0,16 | 1,00 | 0,97 | (0.32 to 3.06) |
| **Infection** | 1 | 59350 | 0,02 | 5 | 44691 | 0,11 | 0,06 | 0,15 | (0.003 to 1.35) |
| **Bleeding** | 0 | 59350 | 0,00 | 2 | 44691 | 0,04 | 0,10 | - | (0.00 to 4.01) |
| **Infiltration** | 14 | 59350 | 0,24 | 9 | 44691 | 0,20 | 0,10 | 1,17 | (0.47 to 3.07) |
| **Other** | 31 | 59350 | 0,52 | 27 | 44691 | 0,60 | 0,58 | 0,86 | (0.50 to 1.51) |
| **Cannulation problem** | 13 | 59350 | 0,22 | 14 | 44691 | 0,31 | 0,36 | 0,70 | (0.30 to 1.60) |
| **Aneurysm** | 2 | 59350 | 0,03 | 6 | 44691 | 0,13 | 0,08 | 0,25 | (0.02 to 1.40) |
|  | **Area puncture** | | | | | | | |  |
|  | **Compli-cations** | **AVF-days** | **Complications /1000 AVF days** | **Compli-cations** | **AVF-days** | **Complications /1000 AVF days** | ***P* value** | **IRR (95% CI)** | |
|  | **Women** | | | **Men** | | |  |  |  |
| **Stenosis** | 6 | 12823 | 0,47 | 5 | 10275 | 0,49 | 1,00 | 0,96 | (0.24 to 3.98) |
| **Thrombosis** | 2 | 12823 | 0,16 | 2 | 10275 | 0,19 | 1,00 | 0,80 | (0.06 to 11.06) |
| **Infection** | 0 | 12823 | 0,00 | 0 | 10275 | 0,00 | - | - | - |
| **Bleeding** | 2 | 12823 | 0,16 | 0 | 10275 | 0,00 | 0,21 | - | - |
| **Infiltration** | 6 | 12823 | 0,47 | 2 | 10275 | 0,19 | 0,30 | 2,40 | (0.43 to 24.35) |
| **Other** | 8 | 12823 | 0,62 | 3 | 10275 | 0,29 | 0,27 | 2,14 | (0.51 to 12.51) |
| **Cannulation problem** | 10 | 12823 | 0,78 | 3 | 10275 | 0,29 | 0,13 | 2,67 | (0.69 to 15.10) |
| **Aneurysm** | 0 | 12823 | 0,00 | 1 | 10275 | 0,10 | 0,26 | - | (0.00 to 31.25) |
|  | **AVF right** | | | **AVF left** | | |  |  |  |
| **Stenosis** | 5 | 8130 | 0,62 | 6 | 14968 | 0,40 | 0,49 | 1,53 | (0.37 to 6.03) |
| **Thrombosis** | 3 | 8130 | 0,37 | 1 | 14968 | 0,07 | 0,14 | 5,52 | (0.44 to 289.96) |
| **Infection** | 0 | 8130 | 0,00 | 0 | 14968 | 0,00 | - | - | - |
| **Bleeding** | 2 | 8130 | 0,25 | 0 | 14968 | 0,00 | 0,06 | - | - |
| **Infiltration** | 6 | 8130 | 0,74 | 2 | 14968 | 0,13 | 0,03 | 5,52 | (0.99 to 55.96) |
| **Other** | 6 | 8130 | 0,74 | 5 | 14968 | 0,33 | 0,20 | 2,21 | (0.56 to 9.15) |
| **Cannulation problem** | 11 | 8130 | 1,35 | 2 | 14968 | 0,13 | **0,0004** | 10,13 | (0.56 to 9.15) |
| **Aneurysm** | 1 | 8130 | 0,12 | 0 | 14968 | 0,00 | 0,17 | - | - |
|  | **Diabetes** | | | **No diabetes** | | |  |  |  |
| **Stenosis** | 6 | 5539 | 1,08 | 5 | 17559 | 0,28 | 0,03 | 3,80 | (0.97 to 15.76) |
| **Thrombosis** | 3 | 5539 | 0,54 | 1 | 17559 | 0,06 | 0,05 | 9,51 | (0.76 to 499.26) |
| **Infection** | 0 | 5539 | 0,00 | 0 | 17559 | 0,00 | - | - | - |
| **Bleeding** | 0 | 5539 | 0,00 | 2 | 17559 | 0,11 | 0,43 | - | (0.00 to 16.88) |
| **Infiltration** | 6 | 5539 | 1,08 | 2 | 17559 | 0,11 | **0,004** | 9,51 | (1.70 to 96.35) |
| **Other** | 5 | 5539 | 0,90 | 6 | 17559 | 0,34 | 0,13 | 2,64 | (0.64 to 10.39) |
| **Cannulation problem** | 10 | 5539 | 1,81 | 3 | 17559 | 0,17 | **0,0001** | 10,57 | (2.72 to 59.75) |
| **Aneurysm** | 1 | 5539 | 0,18 | 0 | 17559 | 0,00 | 0,08 | - | - |
|  | **Age ≥ 70 years old** | | | **Age < 70 years old** | | |  |  |  |
| **Stenosis** | 6 | 11730 | 0,51 | 5 | 11368 | 0,44 | 0,81 | 1,16 | (0.30 to 4.82) |
| **Thrombosis** | 1 | 11730 | 0,09 | 3 | 11368 | 0,26 | 0,36 | 0,32 | (0.006 to 4.02) |
| **Infection** | 0 | 11730 | 0,00 | 0 | 11368 | 0,00 | - | - | - |
| **Bleeding** | 0 | 11730 | 0,00 | 2 | 11368 | 0,18 | 0,15 | - | (0.00 to 5.16) |
| **Infiltration** | 2 | 11730 | 0,17 | 6 | 11368 | 0,53 | 0,17 | 0,32 | (0.03 to 1.81) |
| **Other** | 6 | 11730 | 0,51 | 5 | 11368 | 0,44 | 0,81 | 1,16 | (0.30 to 4.82) |
| **Cannulation problem** | 3 | 11730 | 0,26 | 10 | 11368 | 0,88 | 0,05 | 0,29 | (0.05 to 1.13) |
| **Aneurysm** | 0 | 11730 | 0,00 | 1 | 11368 | 0,09 | 0,31 | - | (0.00 to 37.80) |
| As the significance level is adjusted only p-values <0.008 are bold. Abbreviation: CI confidence interval, IRR incident risk ratio. When IRR is missing, p is based on Incident Rate Difference | | | | | | | | | |
|  | | | | |  |  |  |  |  |

| Table S2. Comparisons and statistical analysis of the number of complications with the different cannulation techniques. | | | | | | | | | | | | | | | | |  |  |  |  |  |  |  |
| --- | --- | --- | --- | --- | --- | --- | --- | --- | --- | --- | --- | --- | --- | --- | --- | --- | --- | --- | --- | --- | --- | --- | --- |
| n=1328 | **No of complic. /1000 AVF days** | **95% CI** | **No of complic /1000 AVF days** | **95% CI** | **IRR** | **95% CI** | **P value** | **IRR MH** | **CI MH** | | **P value** | **IRR MH** | **CI MH** | | **P value** | **IRR MH** | **CI MH** | | **P value** | **IRR MH** | **CI MH** | | **P value** |
|  | **BHs** | | **BHb** | |  |  |  | **Diabetes/No diabetes** | | | | **Women/Men** | | | | **Right/left** | | | | **>70 years old/<70 years old** | | | |
| **Stenosis** | **0.61** | **0.55 to 0.68** | **0.38** | **0.32 to 0.46** | **1.59** | **1.29 to 1.97** | **< 0.001** | **1.61** | **1.31** | **to 1.98** | **< 0.001** | **1.61** | **1.32** | **to 1.98** | **< 0.001** | **1.56** | **1.27** | **to 1.91** | **< 0.001** | **2.82** | **2.31** | **to 3.45** | **< 0.001** |
| **Thrombosis** | 0.12 | 0.09 to 0.15 | 0.10 | 0.07 to 0.14 | 1.13 | 0.73 to 1.77 | 0.58 | 1.13 | 0.74 | to 1.72 | 0.56 | 1.10 | 0.72 | to 1.69 | 0.66 | 1.12 | 0.73 | to 1.71 | 0.60 | 1.16 | 0.76 | to 1.76 | 0.49 |
| **Infection** | 0.06 | 0.04 to 0.09 | 0.04 | 0.02 to 0.07 | 1.55 | 0.79 to 3.21 | 0.18 | 1.56 | 0.81 | to 2.98 | 0.18 | 1.59 | 0.82 | to 3.06 | 0.16 | 1.51 | 0.79 | to 2.89 | 0.21 | 1.63 | 0.85 | to 3.13 | 0.14 |
| **Bleeding** | 0.05 | 0.04 to 0.08 | 0.02 | 0.01 to 0.04 | 2.60 | 1.12 to 7.03 | 0.02 | 2.59 | 1.13 | to 5.93 | 0.02 | 2.36 | 1.06 | to 5.24 | 0.03 | 2.55 | 1.13 | to 5.78 | 0.02 | 2.43 | 1.07 | to 5.48 | 0.03 |
| **Infiltration** | 0.04 | 0.02 to 0.06 | 0.01 | 0.001 to 0.02 | 5.96 | 1.44 to 52.81 | 0.01 | 6.01 | 1.39 | to 26.11 | 0.01 | 5.49 | 1.29 | to 23.33 | 0.01 | 5.91 | 1.33 | to 26.21 | 0.01 | 5.93 | 1.32 | to 26.59 | 0.01 |
| **Other** | 0.48 | 0.42 to 0.54 | 0.36 | 0.3 to 0.43 | 1.35 | 1.08 to 1.69 | 0.01 | 1.35 | 1.09 | to 1.69 | 0.01 | 1.31 | 1.06 | to 1.63 | 0.01 | 1.33 | 1.07 | to 1.65 | 0.01 | 1.32 | 1.07 | to 1.64 | 0.01 |
| **Cannulation difficulty** | **0.18** | **0.14 to 0.22** | **0.07** | **0.04 to 0.1** | **2.57** | **1.61 to 4.24** | **< 0.001** | **2.58** | **1.64** | **to 4.07** | **< 0.001** | **2.46** | **1.55** | **to 3.91** | **< 0.001** | **2.52** | **1.60** | **to 3.97** | **< 0.001** | **2.51** | **1.60** | **to 3.95** | **< 0.001** |
| **Aneurysm** | 0.03 | 0.02 to 0.05 | 0.04 | 0.02 to 0.07 | 0.82 | 0.38 to 1.84 | 0.59 | 0.82 | 0.40 | to 1.69 | 0.59 | 0.78 | 0.39 | to 1.60 | 0.50 | 0.80 | 0.39 | to 1.65 | 0.540 | 0.86 | 0.42 | to 1.76 | 0.67 |
|  | **BHs** | | **RL** | |  |  |  |  |  |  |  |  |  |  |  |  |  |  |  |  |  |  |  |
| **Stenosis** | **0.61** | **0.55 to 0.68** | **1.14** | **0.95 to 1.37** | **0.53** | **0.43 to 0.66** | **< 0.001** | **0.51** | **0.42** | **to 0.64** | **< 0.001** | **0.54** | **0.44** | **to 0.66** | **< 0.001** | **0.53** | **0.43** | **to 0.66** | **< 0.001** | **0.57** | **0.46** | **to 0.70** | **< 0.001** |
| **Thrombosis** | 0.12 | 0.09 to 0.15 | 0.15 | 0.09 to 0.25 | 0.75 | 0.43 to 1.39 | 0.30 | 0.72 | 0.41 | to 1.27 | 0.27 | 0.73 | 0.42 | to 1.27 | 0.27 | 0.75 | 0.43 | to 1.30 | 0.30 | 0.69 | 0.39 | to 1.21 | 0.19 |
| **Infection** | 0.06 | 0.04 to 0.09 | 0.06 | 0.02 to 0.13 | 1.05 | 0.43 to 3.06 | 0.92 | 1.06 | 0.45 | to 2.54 | 0.89 | 1.05 | 0.44 | to 2.51 | 0.91 | 1.05 | 0.44 | to 2.52 | 0.91 | 0.91 | 0.39 | to 2.16 | 0.84 |
| **Bleeding** | 0.05 | 0.04 to 0.08 | 0.02 | 0.002 to 0.07 | 2.84 | 0.72 to 24.60 | 0.13 | 2.98 | 0.71 | to 12.54 | 0.12 | 2.78 | 0.67 | to 11.50 | 0.14 | 2.85 | 0.68 | to 11.95 | 0.13 | 2.98 | 0.76 | to 11.72 | 0.10 |
| **Infiltration** | **0.04** | **0.02 to 0.06** | **0.22** | **0.14 to 0.33** | **0.16** | **0.08 to 0.31** | **< 0.001** | **0.16** | **0.09** | **to 0.30** | **< 0.001** | **0.16** | **0.09** | **to 0.29** | **< 0.001** | **0.16** | **0.09** | **to 0.30** | **< 0.001** | **0.19** | **0.10** | **to 0.34** | **< 0.001** |
| **Other** | 0.48 | 0.42 to 0.54 | 0.56 | 0.42 to 0.72 | 0.86 | 0.65 to 1.17 | 0.31 | 0.85 | 0.64 | to 1.13 | 0.27 | 0.86 | 0.65 | to 1.15 | 0.31 | 0.86 | 0.65 | to 1.15 | 0.31 | 0.87 | 0.65 | to 1.15 | 0.32 |
| **Cannulation difficulty** | 0.18 | 0.14 to 0.22 | 0.26 | 0.17 to 0.38 | 0.68 | 0.44 to 1.09 | 0.08 | 0.65 | 0.43 | to 1.00 | 0.05 | 0.67 | 0.44 | to 1.03 | 0.07 | 0.68 | 0.45 | to 1.05 | 0.08 | 0.69 | 0.45 | to 1.06 | 0.08 |
| **Aneurysm** | 0.03 | 0.02 to 0.05 | 0.08 | 0.03 to 0.15 | 0.42 | 0.17 to 1.12 | 0.04 | 0.42 | 0.18 | to 0.99 | 0.04 | 0.42 | 0.18 | to 0.97 | 0.04 | 0.42 | 0.18 | to 0.97 | 0.04 | 0.35 | 0.15 | to 0.82 | 0.01 |
|  | **BHs** | | **AP** | |  |  |  |  |  |  |  |  |  |  |  |  |  |  |  |  |  |  |  |
| **Stenosis** | 0.61 | 0.55 to 0.68 | 0.48 | 0.24 to 0.85 | 1.29 | 0.71 to 2.60 | 0.41 | 1.31 | 0.72 | to 2.40 | 0.37 | 1.20 | 0.66 | to 2.21 | 0.55 | 0.72 | 2.39 | to 0.78 | 0.38 | 1.33 | 0.73 | to 2.42 | 0.36 |
| **Thrombosis** | 0.12 | 0.09 to 0.15 | 0.17 | 0.05 to 0.44 | 0.66 | 0.25 to 2.52 | 0.42 | 0.68 | 0.25 | to 1.88 | 0.45 | 0.75 | 0.27 | to 2.06 | 0.57 | 0.69 | 0.25 | to 1.90 | 0.47 | 0.62 | 0.23 | to 1.72 | 0.36 |
| **Infection** | 0.06 | 0.04 to 0.09 | 0 | 0 to 0.2 | - | - | 0.24 | - | - | - | 0.24 | - | - | - | 0.24 | - | - | - | 0.22 | - | - | - | 0.26 |
| **Bleeding** | 0.05 | 0.04 to 0.08 | 0.09 | 0.01 to 0.31 | 0.63 | 0.16 to 5.46 | 0.53 | 0.62 | 0.15 | to 2.59 | 0.51 | 0.71 | 0.16 | to 3.08 | 0.65 | 0.64 | 0.15 | to 2.73 | 0.54 | 0.67 | 0.16 | to 2.78 | 0.58 |
| **Infiltration** | **0.04** | **0.02 to 0.06** | **0.35** | **0.15 to 0.68** | **0.10** | **0.04to 0.27** | **< 0.001** | **0.10** | **0.04** | **to 0.24** | **< 0.001** | **0.12** | **0.05** | **to 0.29** | **< 0.001** | **0.11** | **0.05** | **to 0.26** | **< 0.001** | **0.11** | **0.05** | **to 0.25** | **< 0.001** |
| **Other** | 0.48 | 0.42 to 0.54 | 0.48 | 0.24 to 0.85 | 1.01 | 0.55 to 2.05 | 0.98 | 1.02 | 0.55 | to 1.86 | 0.96 | 1.03 | 0.56 | to 1.91 | 0.91 | 1.03 | 0.56 | to 1.89 | 0.92 | 1.02 | 0.55 | to 1.86 | 0.96 |
| **Cannulation difficulty** | **0.18** | **0.14 to 0.22** | **0.56** | **0.3 to 0.96** | **0.31** | **0.18 to 0.61** | **< 0.001** | **0.32** | **0.18** | **to 0.57** | **< 0.001** | **0.36** | **0.20** | **to 0.66** | **< 0.001** | **0.32** | **0.18** | **to 0.58** | **< 0.001** | **0.32** | **0.18** | **to 0.56** | **< 0.001** |
| **Aneurysm** | 0.03 | 0.02 to 0.05 | 0.04 | 0.001 to 0.24 | 0.74 | 0.12 to 30.94 | 0.77 | 0.74 | 0.10 | to 5.65 | 0.77 | 0.75 | 0.10 | to 5.38 | 0.77 | 0.79 | 0.10 | to 6.04 | 0.82 | 0.67 | 0.09 | to 4.99 | 0.69 |
|  | **BHb** | | **RL** | |  |  |  |  |  |  |  |  |  |  |  |  |  |  |  |  |  |  |  |
| **Stenosis** | **0.38** | **0.32 to 0.46** | **1.14** | **0.95 to 1.37** | **0.34** | **0.26 to 0.44** | **< 0.001** | **0.32** | **0.25** | **to 0.41** | **< 0.001** | **0.26** | **0.20** | **to 0.32** | **< 0.001** | **0.35** | **0.27** | **to 0.44** | **< 0.001** | **0.19** | **0.15** | **to 0.24** | **< 0.001** |
| **Thrombosis** | 0.10 | 0.07 to 0.14 | 0.15 | 0.09 to 0.25 | 0.66 | 0.36 to 1.29 | 0.17 | 0.69 | 0.38 | to 1.26 | 0.22 | 0.67 | 0.36 | to 1.24 | 0.20 | 0.66 | 0.36 | to 1.21 | 0.17 | 0.62 | 0.33 | to 1.15 | 0.12 |
| **Infection** | 0.04 | 0.02 to 0.07 | 0.06 | 0.02 to 0.13 | 0.68 | 0.24 to 2.17 | 0.43 | 0.61 | 0.23 | to 1.65 | 0.33 | **0.30** | **0.11** | **to 0.80** | **0.01** | 0.70 | 0.26 | to 1.87 | 0.47 | 0.47 | 0.17 | to 1.27 | 0.13 |
| **Bleeding** | 0.02 | 0.01 to 0.04 | 0.02 | 0.002 to 0.07 | 1.09 | 0.21 to 10.79 | 0.91 | 1.09 | 0.22 | to 5.42 | 0.92 | 1.28 | 0.26 | to 6.32 | 0.76 | 1.16 | 0.24 | to 5.60 | 0.85 | 1.30 | 0.33 | to 5.17 | 0.70 |
| **Infiltration** | **0.01** | **0.001 to 0.02** | **0.22** | **0.14 to 0.33** | **0.03** | **0.003 to 0.11** | **< 0.001** | **0.02** | **0.01** | **to 0.11** | **< 0.001** | **0.03** | **0.01** | **to 0.12** | **< 0.001** | **0.03** | **0.01** | **to 0.11** | **< 0.001** | **0.02** | **0.00** | **to 0.12** | **< 0.0001** |
| **Other** | 0.36 | 0.3 to 0.43 | 0.56 | 0.42 to 0.72 | 0.64 | 0.46 to 0.89 | 0.01 | **0.60** | **0.44** | **to 0.82** | **0.001** | 0.66 | 0.48 | to 0.90 | 0.01 | 0.65 | 0.47 | to 0.88 | 0.01 | 0.71 | 0.52 | to 0.97 | 0.03 |
| **Cannulation difficulty** | **0.07** | **0.04 to 0.1** | **0.26** | **0.17 to 0.38** | **0.27** | **0.15 to 0.48** | **< 0.001** | **0.25** | **0.15** | **to 0.44** | **< 0.001** | **0.27** | **0.15** | **to 0.48** | **< 0.001** | **0.28** | **0.16** | **to 0.48** | **< 0.001** | **0.30** | **0.17** | **to 0.51** | **< 0.001** |
| **Aneurysm** | 0.04 | 0.02 to 0.07 | 0.08 | 0.03 to 0.15 | 0.51 | 0.20 to 1.41 | 0.12 | 0.51 | 0.21 | to 1.23 | 0.13 | 0.53 | 0.22 | to 1.25 | 0.14 | 0.51 | 0.21 | to 1.22 | 0.12 | 0.43 | 0.18 | to 1.04 | 0.05 |
|  | **AP** | | **BHb** | |  |  |  |  |  |  |  |  |  |  |  |  |  |  |  |  |  |  |  |
| **Stenosis** | 0.48 | 0.24 to 0.85 | 0.38 | 0.32 to 0.46 | 1.24 | 0.60 to 2.29 | 0.49 | 1.22 | 0.66 | to 2.27 | 0.52 | 1.25 | 0.67 | to 2.35 | 0.48 | 1.14 | 0.62 | to 2.12 | 0.67 | 2.10 | 1.14 | to 3.89 | 0.02 |
| **Thrombosis** | 0.17 | 0.05 to 0.44 | 0.10 | 0.07 to 0.14 | 1.70 | 0.44 to 4.75 | 0.31 | 1.70 | 0.60 | to 4.81 | 0.31 | 1.88 | 0.65 | to 5.48 | 0.24 | 1.72 | 0.59 | to 5.00 | 0.31 | 1.82 | 0.65 | to 5.10 | 0.25 |
| **Infection** | 0 | 0 to 0.2 | 0.04 | 0.02 to 0.07 | 0.00 | 0.00 to 4.73 | 0.34 | - | - | - | 0.34 | - | - | - | 0.42 | - | - | - | 0.34 | - | - | - | 0.40 |
| **Bleeding** | 0.09 | 0.01 to 0.31 | 0.02 | 0.01 to 0.04 | 4.12 | 0.42 to 21.63 | 0.06 | 4.11 | 0.86 | to 19.68 | 0.06 | 2.19 | 0.43 | to 11.08 | 0.33 | 3.52 | 0.68 | to 18.18 | 0.11 | 3.25 | 0.74 | to 14.25 | 0.10 |
| **Infiltration** | **0.35** | **0.15 to 0.68** | **0.01** | **0.001 to 0.02** | **57.66** | **11.51 to 557.34** | **< 0.001** | **56.41** | **11.85** | **to 268.6** | **< 0.001** | **39.22** | **7.14** | **to 215.3** | **< 0.001** | **66.54** | **10.18** | **to 434.8** | **< 0.001** | **74.09** | **14.09** | **to 389.7** | **< 0.001** |
| **Other** | 0.48 | 0.24 to 0.85 | 0.36 | 0.3 to 0.43 | 1.33 | 0.65 to 2.47 | 0.36 | 1.32 | 0.71 | to 2.45 | 0.38 | 1.07 | 0.57 | to 2.01 | 0.84 | 1.26 | 0.67 | to 2.34 | 0.47 | 1.21 | 0.65 | to 2.24 | 0.54 |
| **Cannulation difficulty** | **0.56** | **0.3 to 0.96** | **0.07** | **0.04 to 0.1** | **8.15** | **3.79 to 16.77** | **< 0.001** | **8.11** | **4.09** | **to 16.08** | **< 0.001** | **7.61** | **3.51** | **to 16.48** | **< 0.001** | **7.37** | **3.59** | **to 15.13** | **< 0.001** | **7.35** | **3.83** | **to 14.13** | **< 0.001** |
| **Aneurysm** | 0.04 | 0.001 to 0.24 | 0.04 | 0.02 to 0.07 | 1.11 | 0.03 to 7.38 | 0.92 | 1.10 | 0.14 | to 8.51 | 0.92 | 0.86 | 0.13 | to 5.81 | 0.87 | 1.02 | 0.13 | to 8.25 | 0.98 | 1.23 | 0.16 | to 9.15 | 0.98 |
|  | **AP** | | **RL** | |  |  |  |  |  |  |  |  |  |  |  |  |  |  |  |  |  |  |  |
| **Stenosis** | **0.48** | **0.24 to 0.85** | **1.14** | **0.95 to 1.37** | **0.42** | **0.20 to 0.77** | **0.004** | **0.41** | **0.22** | **to 0.77** | **0.004** | 0.43 | 0.22 | to 0.86 | 0.01 | **0.41** | **0.22** | **to 0.76** | **0.004** | 0.43 | 0.23 | to 0.80 | 0.01 |
| **Thrombosis** | 0.17 | 0.05 to 0.44 | 0.15 | 0.09 to 0.25 | 1.13 | 0.27 to 3.49 | 0.83 | 1.10 | 0.34 | to 3.58 | 0.87 | 0.85 | 0.30 | to 2.43 | 0.76 | 1.07 | 0.35 | to 3.26 | 0.91 | 1.11 | 0.37 | to 3.36 | 0.85 |
| **Infection** | 0 | 0 to 0.2 | 0.06 | 0.02 to 0.13 | 0.00 | 0.00 to 3.83 | 0.25 | - | - | - | 0.24 | - | - | - | 0.15 | - | - | - | 0.22 | - | - | - | 0.23 |
| **Bleeding** | 0.09 | 0.01 to 0.31 | 0.02 | 0.002 to 0.07 | 4.50 | 0.33 to 62.14 | 0.10 | 5.09 | 0.72 | to 36.10 | 0.07 | 2.36 | 0.33 | to 16.74 | 0.15 | 4.05 | 0.53 | to 31.16 | 0.15 | 3.93 | 0.55 | to 27.91 | 0.14 |
| **Infiltration** | 0.35 | 0.15 to 0.68 | 0.22 | 0.14 to 0.33 | 1.57 | 0.61 to 3.63 | 0.27 | 1.36 | 0.58 | to 3.17 | 0.47 | 1.31 | 0.57 | to 3.01 | 0.53 | 1.53 | 0.67 | to 3.51 | 0.31 | 1.56 | 0.69 | to 3.53 | 0.28 |
| **Other** | 0.48 | 0.24 to 0.85 | 0.56 | 0.42 to 0.72 | 0.85 | 0.40 to 1.64 | 0.63 | 0.79 | 0.41 | to 1.52 | 0.48 | 0.90 | 0.45 | to 1.79 | 0.76 | 0.87 | 0.45 | to 1.67 | 0.67 | 0.85 | 0.45 | to 1.62 | 0.62 |
| **Cannulation difficulty** | 0.56 | 0.3 to 0.96 | 0.26 | 0.17 to 0.38 | 2.17 | 1.03 to 4.35 | 0.02 | 1.74 | 0.87 | to 3.45 | 0.11 | 1.73 | 0.87 | to 3.44 | 0.11 | 2.04 | 1.03 | to 4.04 | 0.04 | 2.10 | 1.08 | to 4.10 | 0.03 |
| **Aneurysm** | 0.04 | 0.001 to 0.24 | 0.08 | 0.03 to 0.15 | 0.56 | 0.01 to 4.20 | 0.58 | 0.51 | 0.05 | to 4.82 | 0.55 | 0.76 | 0.10 | to 5.70 | 0.79 | 0.56 | 0.07 | to 4.90 | 0.60 | 0.52 | 0.06 | to 4.18 | 0.53 |
| As the significance level is adjusted only p-values <0.008 are bold. Abbreviation: CI confidence intervall. IRR incidens risk ratio MH. Mantel-Haenszel; CI. confidence interval; IRR. incidence risk ratio; BHs. buttonhole sharp; BHb. buttonhole blunt; RL. rope ladder; AP. area puncture. | | | | | | | | | | | | | | | | | | | | | | | |
